# Absent Bell’s phenomenon in patients with thyroid eye disease

**DOI:** 10.1186/s12886-021-02107-x

**Published:** 2021-10-11

**Authors:** Hyun Woo Chung, Hwa Lee, Sehyun Baek

**Affiliations:** 1 Department of Ophthalmology, Ansan Hospital, Korea University College of Medicine, 123 Jeokkumro, Ansan-shi, Kyunggi-do 15355 Republic of Korea; 2 Department of Ophthalmology, Guro Hospital, Korea University College of Medicine, 148 Gurodongro, Seoul, 08308 Republic of Korea; 3 Department of Ophthalmology, Korea University College of Medicine, Seoul, South Korea

**Keywords:** Bell’s phenomeon, Fibrosis, Inferior rectus muscle, Thryoid eye disease

## Abstract

**Background:**

To investigate the incidence of absent Bell’s phenomenon (BP) and the relationship between absent BP and inferior rectus muscle hypertrophy and other clinical features in patients with thyroid eye disease (TED).

**Methods:**

A total of 104 patients who were first diagnosed with TED between January and December 2014 were included. Inferior rectus muscle area and associations with clinical features of TED and thyroid function test including thyroid specific antibodies were compared between patients with TED with and without BP. The volume of the inferior rectus muscle was calculated by adding up all the cross-sectional areas measured on sagittal CT images.

**Results:**

Among the 104 patients, 14 had absent BP (13.5%), 12 with bilateral and two with unilateral. There was no significant difference in thyroid function test, presence of TSIs, exophthalmos, or volume of inferior rectus muscle measured in CT scans (*P* > 0.05). Incidence of diplopia, elevation limitation, and upper eyelid retraction were risk factors of absent BP in TED patients (by logistic regression analysis, *P* < 0.05).

**Conclusions:**

Inferior rectus muscle hypertrophy was not the cause of absent BP in TED patients. Fibrosis and tightening of the inferior rectus muscle, lower eyelid, and surrounding orbital tissues, rather than inferior rectus muscle hypertrophy, might be related to absent BP in TED patients.

## Background

The clinical features of thyroid eye disease (TED) consist of a variable combination of eyelid retraction, eyelid swelling, proptosis, impaired ocular motility, keratitis, exposure keratopathy, and optic nerve compression [1, 2]. One of the leading causes of ocular surface damage in TED is dry eye syndrome [3]. Factors considered to cause dry eye in TED include exophthalmos, increasing palpebral fissure height, lagophthalmos, and reduced tear production [4, 5].

Sight-threatening corneal ulceration is far less common than dysthyroid optic neuropathy and presents as corneal staining, sometimes with thinning and very occasionally with corneal perforation [6]. Recently reported incidence of corneal ulcer in thyroid eye disease patients was 1.3% [7]. In addition, corneal exposure and ulceration could take place when the eyelids are incompletely closed due to lagophthalmos and there is no Bell’s phenomenon (BP). Hence, absent BP with lagophthalmos increases the risk of corneal damage.

Absence of BP was first reported by Charles Bell in 1823 as an upward deviation of the eye during forcible eyelid closure with a lower motor neuron defect of the facial nerve [8]. Although the physiological mechanism has not been fully explained, normal function of extraocular muscles is essential for this sign. Although BP is absent in 10% individuals, it is more likely to be lost in TED patient with very tight inferior rectus muscle limiting upward movement of the eyeball [9]. However, the exact incidence of loss of this reflex and whether BP is correlated with inferior right rectus muscle hypertrophy, tight rectus muscle, or other clinical factors have not been studied.

The purpose of this study is to investigate the incidence of absent BP and the relationship between absent BP and inferior rectus muscle hypertrophy and other clinical features in patients with TED.

## Methods

After receiving approval from the Institutional Review Board at Korea University College of Medicine Guro and Ansan Hospital, 104 patients who were diagnosed with TED in accordance with the diagnostic criteria of the American Academy of Ophthalmology, from January to December 2014, were enrolled in this cross-sectional study [10]. The study was conducted in accordance with the Declaration of Helsinki. Patients who had undergone eyelid surgery, radiation therapy, decompression, or strabismus surgery were excluded. Patients who had an incomplete set of CT (computed tomography) images were also excluded.

Laboratory measurements for TSH (Thyroid Stimulating Hormone), FT4(Free T4), T3, and thyroid specific antibodies, including thyroid peroxidase antibodies (TPOAbs) and thyroid-stimulating immunoglobulin (TSI), were assessed.

The following clinical features were investigated: age, sex, thyroid status and systemic treatment, best-corrected visual acuity (BCVA), color vision, lid swelling, upper and lower eyelid retraction, lid lag, keratopathy, lagophthalmos, exophthalmos with a Hertel exophthalmometer (Oculus; Oculus Optik Geraet, Wetzlar, Germany), diplopia score according to the Gorman, [11] abduction, adduction, elevation, depression, Clinical Activity Score (CAS), [12] and modified NOSPECS classification [13]. Upper eyelid retraction was defined as the upper eyelid at or above the superior limbus in primary position without frontalis muscle contraction, while lower eyelid retraction was position of the lower eyelid below the inferior limbus in primary position [14].

Symptoms of diplopia were recorded based on Bahn–Gorman progression: 0 as no diplopia, 1 as intermittent diplopia (present with fatigue), 2 as inconstant diplopia (with vertical or horizontal gaze), 3 as constant diplopia in straight gaze that is correctable with prisms, and 4 as constant diplopia that is not correctable with prisms [15]. The degree of elevation limitation as a sign of inferior rectus involvement was graded on a scale of − 1 to − 4; − 4 indicates no elevation movement, − 3 indicates 25% movement remains, − 2 indicates 50% movement remains, and − 1 indicates 75% movement remains. CAS consisted of the following seven items: retrobulbar pain with movement and/or rest, redness of the eyelids and/or conjunctivae, swelling of the caruncle, swelling of the eyelids, or chemosis. A CAS score greater than 4 was considered active disease [9].

**Fig. 1:**
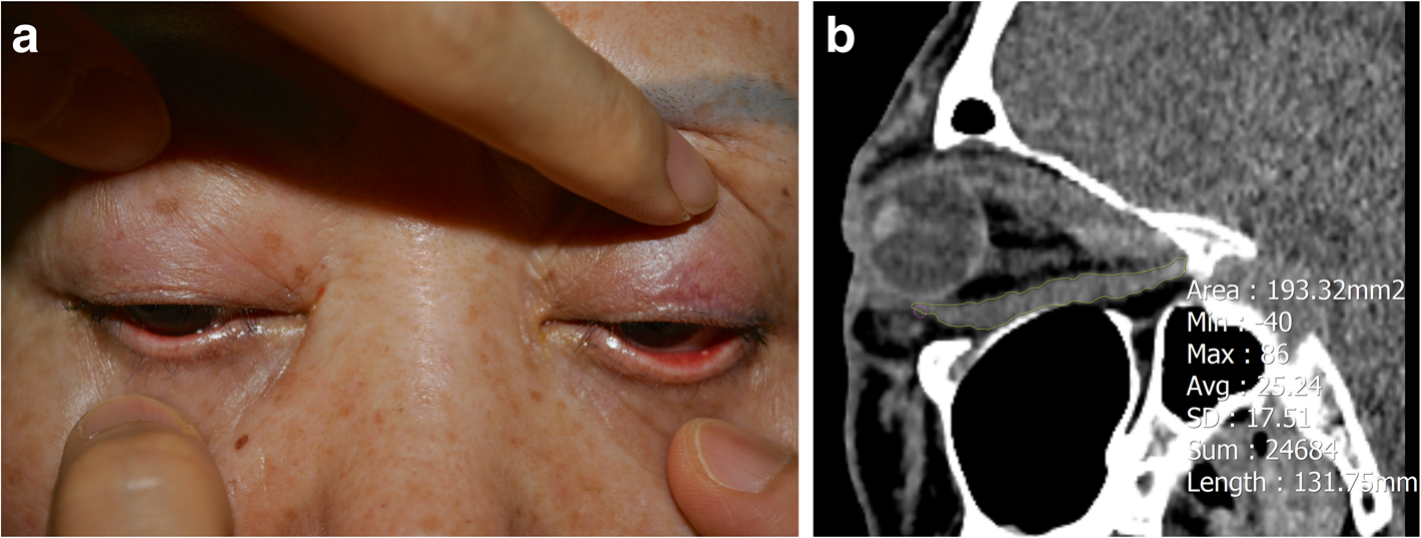
**A** Bell’s phenomenon is assessed by gently lifting each upper lid manually while the patient’s eyes are closed. The examination shows absent BP in both eyes. **B** Measurement of cross-sectional areas of inferior rectus muscle on sagittal CT images

BP was assessed by gently lifting each upper lid manually while the patients attempted gentle closure while observing closely whether upward rotation takes place. If there was no upward rotation, then it was regarded absent BP (Fig. 1A). Non-contrast orbital CT was performed on a 128 row multi-detector CT (Ingenuity Core 128, Philips Healthcare, Cleveland, USA) with 3 mm thickness of axial, coronal, and sagittal images. The cross-sectional areas of the inferior rectus muscle (IR) were measured by tracing outlines of each tissue on sagittal CT images using a PACS system (PiViewSTAR, INFINITT, Korea) (Fig. 1B). The volume of the IR was calculated by adding up all the cross-sectional volumes in the same manner as previous studies [16, 17].

Presence of BP was investigated in control group who visited the clinic and diagnosed with dry eye syndrome, age and sex matched as TED patients.

For statistical analyses, SPSS software version 18.0 (SPSS Inc., Chicago, IL) was used. To analyze differences in the independent variables, Fisher’s exact test was used. To analyze differences in continuous variables, Mann-Whitney U-test was used in the case of an abnormal distribution. We used multivariate logistic regression analysis to assess absent BP risk factors including presence of diplopia, elevation limitation, and the incidence of upper eyelid retraction were included. *P*-values less than 0.05 were considered statistically significant.

## Results

During the enrollment period, a total of 104 patients were diagnosed with TED at Korea University Guro Hospital and Korea University Ansan Hospital. Among them, 14 patients showed negative BP (13.5%), 12 were bilateral, and two were unilateral. Patient demographics and thyroid dysfunctions are shown in Table 1. There was a female predominance with either absent or positive BP. The mean age was higher in absent BP patients (54.0 ± 11.9 years) than in positive BP patients (44.1 ± 14.9 years), but the difference was not significant (*P* = 0.15, by Mann–Whitney U test). Thyroid dysfunction was noted in 28% and positive TSI was noted in 34% of patients with absent BP. (Table 1) The differences in thyroid dysfunction, TPOAbs, TSI, thyroid treatment including antithyroid medication and radioactive iodine between patients with positive BP and patients with negative BP were not statistically significant (*P* > 0.05).

**Table 1:** Characteristics of thyroid eye disease patients according to presence of Bell’s phenomenon

	Bell’s phenomenon +	Bell’s phenomenon -	*p*-value
Patients [N (%)]	90 (86.5)	14 (13.5)	
Age (years)	44.1 ± 14.9 (20 ~ 66)	54.0 ± 11.9 (19 ~ 66)	0.15
Sex [N (%)]			
Male	28 (31)	2 (14)	
Female	62 (69)	12 (86)	0.196
Disease duration (months)	12 ± 14.4	23 ± 9.9	0.23
Thyroid dysfunction	25 (28%)	4 (29%)	0.645
Antithyroid medications	24 (27%)	4 (29%)	0.607
RAI	4 (4%)	2 (14%)	0.225
TPO Abs	11 (12%)	2 (14%)	0.828
TSI	31 (34%)	5 (36%)	0.926

By Fisher’s exact test and Mann-Whitney U-test

*Abbreviations*: *RAI* Radioactive iodine, *TPO* Abs Thyroid peroxidase antibodies, *TSI* Thyroid-stimulating immunoglobulin

The mean exophthalmometry values were 14.7/15.8 mm in absent BP patients (right/left, range 10.0–28.0) and 16.9/16.6 mm in positive BP patients (range 13.0–21.0)(*p* = 0.179, 0.504) (Table 2). Clinically significant active disease, defined as a CAS > 4/10, was not present in negative BP patients. The CAS score was the same at 1.2 in BP absent and positive patients. The mean NOSPECS score was 2.0 in absent BP patients and 2.6 in positive BP patients.

**Table 2:** Analysis of risk factors in clinical characteristics for absent Bell’s phenomenon

	Bell’s phenomenon +	Bell’s phenomeon -	*p*-value
Exophthalmometry (Rt/Lt)	16.9/16.6	14.7/15.8	0.179/0.504
CAS score (Rt/Lt)	1.2 ± 1.2	1.2 ± 0.8	0.895
Active phase	10 (11.1%)	0 (0%)	0.190
NOSPECS score	2.6 ± 2.3	2.0 ± 1.6	0.554
Lagophthalmos	14 (16%)	4 (40%)	0.231
Presence of diplopia	16 (18%)	7 (50%)	0.007*
Bahn–Gorman score	0.4	1.4	0.341
Elevation limitation (Rt/Lt)	14/16 (16%/18%)	6/6 (43%/43%)	0.016/0.033*
Eyelid retraction (Rt/Lt)	Upper 12/14 (13%/16%)	9/9 (64%/64%)	0.000/0.000*
	Lower 10/16 (11%/18%)	0/3 (0%/21%)	0.190/0.742
Volume of inferior rectus muscle (mm^3^)(Rt/Lt)	1148.7 / 1069.9	1458.4 / 1389.5	0.311/0.190

by Multivariate logistic regression analysis, *p*-value < 0.05*

Presence of diplopia was 50% in absent BP patients and 18% in positive BP patients (*P* = 0.007 by logistic regression analysis, Odds ratio 7.3 95%CI). Elevation limitation was 43%/43% (right/left) in absent BP patients and 16%/18% in positive BP patients (right/left)(*P* = 0.016/0.033, Odds ratio 5.8/4.6 95%CI). The incidence of upper eyelid retraction was 64%/64% (right/left) in absent BP and 13%/16% in positive BP patients (*P* = 0.000/0.000, Odds ratio 21.1/16.7 95%CI).

Mean volume of inferior rectus muscle was 1148.7 ± 198.6 / 1069.9 ± 181.1 mm^3^ (right/left) in positive BP patients and 1458.4 ± 162.9 / 1389.5 ± 179.3 mm^3^ in absent BP patients (*P* = 0.311/0.190).

In control group, dry eye syndrome patients with age and sex matched as TED patients, incidence rate of absent BP was 11.5% (12 patients out of 104 patients). In absent BP group, 7 patients were male (58.3%). The mean age was higher in BP absent patients (51.3 ± 10.4 years) than in BP positive patients (44.6 ± 15.3 years). The difference of the absent BP incidence was not statistically significant (*P* = 0.675).

## Discussion

Charles Bell encountered many cases of unilateral paralysis of the facial muscles and noted that the eyeball on the paralyzed side invariably rotates upward when the patient tries to close her/his eyelids [18]. This palpebral-oculogyric reflex, which is more noticeable when eyelid closure is incomplete, is known as BP. Although this reflex is absent in 10% of individuals, it is more likely to be absent in TED patient who has a very tight inferior rectus [9]. However the incidence of absent BP in TED patients was 13.5% and not significantly higher to that of the control group in our study (11.5%).

TED is an immune-mediated inflammatory disorder that causes expansion of the extraocular muscles and orbital fat from edema with deposition of collagen and glycosaminoglycans [19, 20]. The typical course of a TED patient without specific treatment is initial active progressive phase, followed by a phase of spontaneous slow improvement described by Rundle [21].

After regression of the inflammatory process, fibrosis may develop; affected tissues may display traits such as proptosis; and eyelid retraction and chronic dysfunction of extraocular muscles might persist and not return to their previous normal functioning state.

In our study, there were none of the patients with absent BP had active TED. Although mean volume of inferior rectus muscle in absent BP group was larger than that of positive BP group, the difference was not statistically significant. However, incidence of diplopia, elevation limitation, and upper eyelid retraction in TED patients without BP were significantly different from those with BP. These suggest that absent BP is more likely to be related to fibrosis and tightening of the orbital tissues rather than active inflammation and orbital tissue and rectus muscle hypertrophy.

TED patients can be divided into four groups: no fat volume or muscle volume increase, only fat volume increase, only muscle volume increase, and both fat and muscle volume increases [22]. Increase in muscle volume was present in the largest group and was related to older age, more proptosis, and reduced duction values [23]. Although extraocular muscle volume could have a relationship with duction limitation and more proptosis, fibrosis and tightening of the extraocular muscle and eyelid retractors rather than volume increase itself could exert more influence on extraocular movement and the normal protective BP.

Eyelid retraction can involve the upper or lower eyelid and has been suggested as the most common sign and one of the prime diagnostic criteria for TED [15]. The cause of upper eyelid retraction is multifactorial as follows; increased sympathetic tone in Müller’s muscle, levator muscle fiber enlargement, levator muscle contracture or fibrosis, relative or absolute exophthalmos, or scarring and/or inflammation of the septum and anterior lamella [24, 25]. In addition, tight restriction of the inferior rectus muscle leads to upper eyelid retraction, regardless of upper eyelid pathology [15]. It causes resultant increase in tone of the superior rectus and levator muscle [26].

In our study, the absent BP patients were more likely have elevation limitation and upper eyelid retraction and this could be explained by the fixation duress. Fixation duress was presumed to play an additional role in the pathogenesis of upper eyelid retraction in patients with profound restriction of elevation from the primary position and inferior rectus muscle restriction [26–28]. Upper eyelid retraction clinically apparent when fixating in the primary position or attempting upgaze resolved on downgaze. Longstanding contraction of the levator muscle due to this fixation duress may result in shortening and contracture of the levator muscle that does not readily resolve on relief of the inferior rectus muscle restriction [29].

Limitation of this study is the presence of weak but positive BP. Patients were classified roughly into 2 groups, namely presence or absence of BP. The group designated as positive BP is suspected to have included many patients with weak but positive BP, probably resulting into no statistically significant difference in some factors between the groups. To measure the amount of BP and analyze the influential factors may provide more information. Another limitation is the measurement method of the IR volume. There has been various measurement method including calculating cross-sectional area in coronal Magnetic Resonance (MR) imaging using maximum diameters approximately 1 cm behind posterior pole of globe [30] and cross-sectional areas of the IR by tracing outlines of each tissue on sagittal CT and MR images [16, 17]. The latter method was used in this study. Although no consensus exists regarding the quantification of EOM size in TED but various volumetric analysis might provide another information.

The combination of eyelid retraction and proptosis in TED increases corneal exposure and may lead to symptoms of irritation, photophobia, secondary epiphora, and blurred vision [31]. Corneal ulceration arises from lagophthalmos and corneal exposure due to proptosis, lower eyelid retraction, and/or poor levator function, usually accompanied by a tight inferior rectus [32]. Although the incidence of absent BP in TED patients is not higher than that of control group in our study, it has to be investigated further if corneal exposure or ulceration occur more often in patients having both absent BP and lagophthalmos vs. only absent BP.

The test for the presence of BP is not a routinely examined in clinical settings, so more attention should be paid to it especially in patients with lagophthalmos. We recommend that presence/ absence of BP should be carefully performed in patients with TED especially those with lagophthalmos.

## Conclusion

In conclusion, we reported the prevalence (13.5%) of absent BP in 104 Korean patients with TED. Inferior rectus muscle hypertrophy was not the cause of absent BP in TED patients. Instead, fibrosis and tightening of the inferior rectus muscle, eyelid, and surrounding orbital tissues might be related to absent BP in TED patients.

## Data Availability

The datasets used and analyzed during the current study are available from the corresponding author on reasonable request.

## Abbreviations

BP: Bell’s phenomenon
CAS: Clinical Activity Score
FT4: Free T4
IR: Inferior rectus muscle
TED: Thyroid eye disease
TPOAbs: Thyroid peroxidase antibodies
TSH: Thyroid stimulating hormone
TSI: Thyroid-stimulating immunoglobulin
